# Intensity Control During Block-Periodized High-Intensity Training: Heart Rate and Lactate Concentration During Three Annual Seasons in World-Class Cross-Country Skiers

**DOI:** 10.3389/fspor.2020.549407

**Published:** 2020-10-21

**Authors:** Trine Karlsen, Guro Strøm Solli, Svein Tore Samdal, Øyvind Sandbakk

**Affiliations:** ^1^Faculty of Nursing and Health Sciences, Nord University, Bodø, Norway; ^2^Department of Sports Science and Physical Education, Faculty of Education and Arts, Nord University, Bodø, Norway; ^3^Department of Neuromedicine and Movement Science, Centre for Elite Sports Research, NTNU, Norwegian University of Science and Technology, Trondheim, Norway; ^4^The Norwegian Ski Association, Oslo, Norway

**Keywords:** endurance training, cross-country skiing, periodization, XC skiing, HIT, lactate

## Abstract

**Purpose:** To describe heart rate (HR) and blood lactate (Bla^−^) responses during high-intensity interval training (HIT) in a long-term block-periodized HIT regimen in world-class cross-country (XC) skiers.

**Methods:** Data were collected in 14 world-class female XC skiers (aged 25 ± 5 years; body mass, 60.4 ± 6.5 kg; and maximal HR, 194 ± 8 beats · min^−1^) throughout three entire seasons. The HR and Bla^−^ values were determined at the end of 572 intervals performed during 63 sessions and 17 HIT blocks utilizing different exercise modes: running, running with poles, and skiing (on-snow and roller ski) with classic and skating techniques.

**Results:** The mean HR was 91 ± 3% of HR_max_ with a corresponding Bla^−^ of 7.3 ± 2.1 mmol · L^−1^. The average HR and Bla^−^ values were relatively similar across the different exercise modes, except for a lower HR (~90 vs. 92% of HR_max_) for on-snow and roller ski classical skiing and lower Bla^−^ values (5.9 vs. 7.0–7.8 mmol · L^−1^) for on-snow classical skiing compared to the other modes, both *P* < 0.05. An increase in HR and Bla^−^ was observed from interval working periods 1 to 3 (90–92% of HR_max_ and 6.5–7.7 mmol · L^−1^) and further from 3 to 5 (92–93% of HR_max_ and 7.7–9.0 mmol · L^−1^), all *P* < 0.05.

**Conclusions:** We describe long-term use of HIT-block periodization among world-class XC skiers who achieved target HR and Bla^−^ levels in all six exercise modes employed. According to athletes and coaches, the key to successful blocks was intensity control to allow for high-quality HIT sessions throughout the entire HIT block.

## Introduction

Cross-country (XC) skiing is a demanding endurance sport, where top athletes have achieved some of the highest maximal oxygen uptake (VO_2max_) levels ever reported in scientific literature. To reach VO_2max_ values >85 mL · kg^−1^ · min^−1^ for men and >70 mL · kg^−1^ · min^−1^ for women (Ingjer, 1991; Losnegard and Hallén, 2014; Tønnessen et al., 2014, 2015; Sandbakk et al., 2016), XC skiers' training primarily targets aerobic endurance capacity. The typical training model among XC skiers includes 700–850 h per year of endurance training, distributed as 90% low (LIT), 4–5% as moderate (MIT), and 5–8% as high-intensity training (HIT) (Sandbakk and Holmberg, 2017). HIT sessions usually make up only 5–8% of the annual training time and ~20% of the total annual number of endurance sessions (Seiler, 2010; Tønnessen et al., 2014; Stöggl and Sperlich, 2015; Sandbakk et al., 2016; Solli et al., 2017) and are regarded vital in eliciting physiological and performance gains (Laursen and Jenkins, 2002; Iaia et al., 2009; Buchheit and Laursen, 2013a,b; Gunnarsson et al., 2013). It has been argued that an increased volume and/or frequency of HIT would be beneficial for the further development of elite endurance athletes (Laursen, 2010).

Most studies have reported that the periodization of HIT vs. MIT and LIT in endurance sports is achieved via the traditional periodization model (TRAD) (Matwejew, 1975; Issurin, 2008; Tønnessen et al., 2014), characterized by a mixed focus on LIT, MIT, and HIT in all periods but with a gradual progression from high training volume to higher training intensity as the competition period approaches. As an alternative, it has been argued that a more effective way of organizing endurance training is to include defined blocks of increased focus on specific intensities (Issurin, 2008, 2010, 2016, 2018), such as block periodization (BP) of HIT. BP aims to direct highly concentrated HIT stimuli to induce a beneficial physiological impact and appropriate hormonal response to optimize the subsequent adaptations (Issurin, 2018). Positive short-term effects of using BP of HIT to augment training responses have been shown (Breil et al., 2010; Støren et al., 2012; Wahl et al., 2013; Clark et al., 2014; Rønnestad et al., 2017), whereas a recent study of junior XC skiers reported no beneficial effects of BP over TRAD (McGawley et al., 2017). In addition, a meta-analysis concluded that BP of HIT is an adequate, alternative strategy with potentially higher training effects than TRAD for trained to well-trained athletes (Mølmen et al., 2019). However, all these studies have compared the different periodization models by matching the overall HIT volume, whereas in practice, athletes also use BP of HIT to increase the overall HIT stimuli (Solli et al., 2019).

Most previous studies on BP of HIT have been limited by short intervention periods (4–12 weeks). However, one unique study of two comparably successful seasons in the world's best XC skier has compared detailed microperiodization, mesoperiodization, and macroperiodization of BP of HIT with TRAD, illustrating how the organization of the BP of HIT can be utilized in connection with intensified training in elite endurance athletes (Solli et al., 2019). Despite similar endurance training loads, twice as many HIT sessions were performed during the BP year than the TRAD year. This was achieved by organizing 45% of the annual HIT sessions in seven major HIT blocks lasting from 7 to 11 days. Each block included 8–13 HIT sessions employing various exercise modes (running, running with poles, as well as roller ski and on-snow ski skating and classic techniques). The athlete was a Norwegian national team member and the HIT training organization representative for the entire national team, with yearly BP of HIT cycles systematically monitored in the 2003–2006 seasons.

By using the unique dataset from the interval session monitoring of extensive HIT blocks, the primary aim of this article is to describe physiological responses [i.e., blood lactate (Bla^−^) and heart rate (HR) values] of HIT intervals during a long-term BP regimen among world-class female XC skiers. Our secondary aim was to investigate the physiological responses of HIT sessions between different exercise modes and with the development of intervals within these sessions.

## Materials and Methods

### Participants

Data were collected during the 2003–2006 seasons in 14 Norwegian world-class female XC skiers (aged 25 ± 5 years; body mass, 60.4 ± 6.5 kg), including four world- and Olympic-medal winners. During the three investigated seasons, these athletes achieved 13, 18, and 17 individual podium places, respectively, in world cup races and a total of eight medals in the 2006 Olympic Games and the 2005 World Championship. Data were collected as part of routine testing during 17 training camps and 63 HIT sessions (majority with 5 × 4 min intervals), with Bla^−^ and HR values determined at the end of 572 distinct intervals.

### Ethical Statement

This article reports routine physiological testing and training in healthy athletes. Testing was voluntary for the athletes and undertaken for physiological monitoring, not for scientific purposes. Written informed consents for publication of the data were given by the athletes or next of kin in 2020. The Regional Committee for Medical and Health Research Ethics waives the requirement for ethical approval for such studies as publication of these data does not fall under the law for medical and health research. Therefore, the ethics of the publication is done according to the institutional requirements.

### Training Regimen

**Figure 1:**
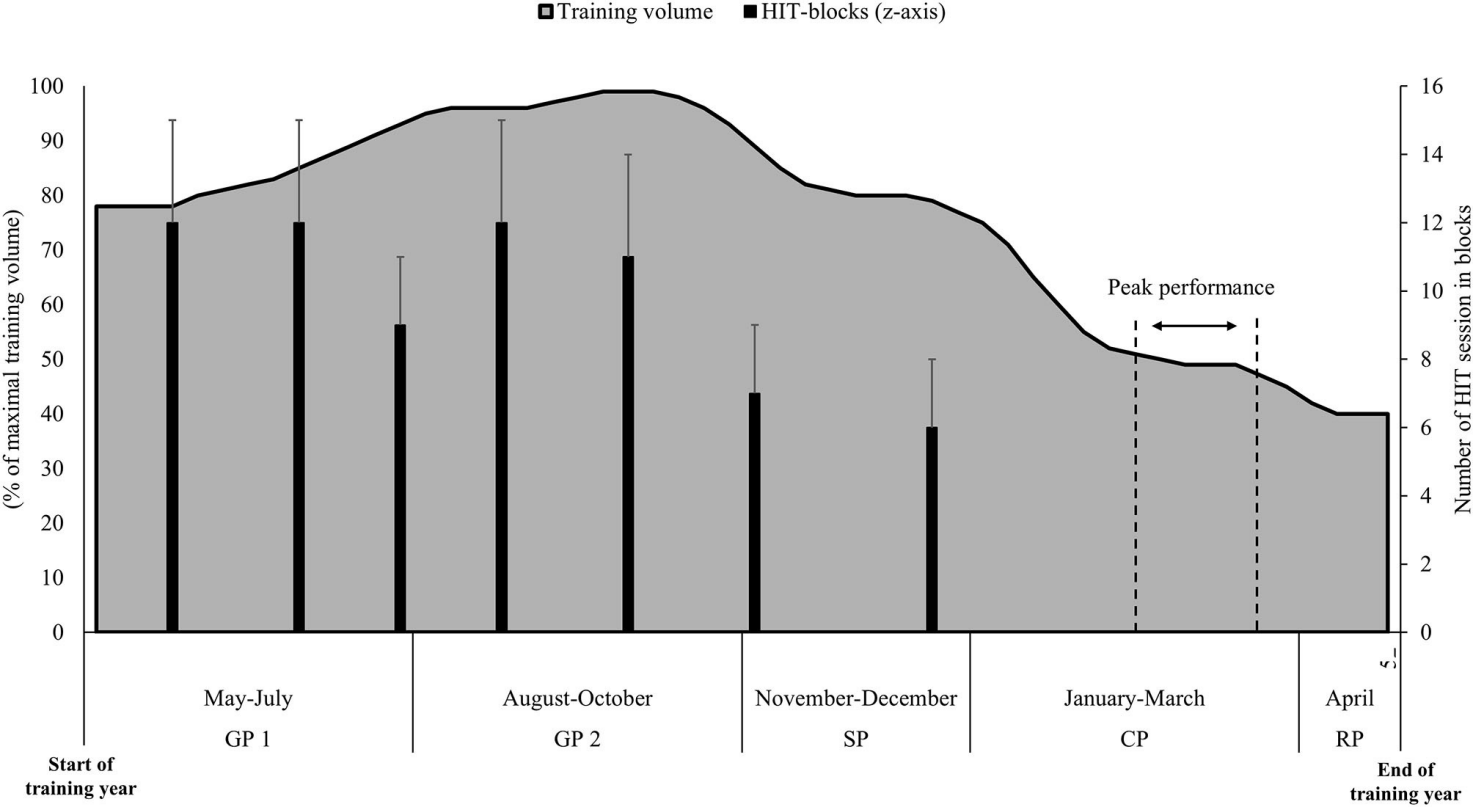
Illustration of the high-intensity training blocks, as well as the average monthly training volume and peaking of performance (Olympics or world-championships) in the different phases (GP, general preparation period; SP, specific preparation period; CP, competition period; RP, regeneration period) of the annual training cycle among the world-class female cross-country skiers examined in the current study.

Before the start of the BP regimen, training was organized according to a traditional periodization model of endurance training, including an even distribution of two to three HIT sessions per week throughout the annual season. The systematic use of BP of HIT was implemented throughout three entire seasons as part of a training regimen with an overall amount of 650–850 annual training hours. Initially, most of the team's athletes were young and had a VO_2max_ clearly below the world-class level. Therefore, the aim of the training strategy was to accelerate the VO_2max_ development by increasing the amount of HIT by using BP. Using the modified session goal approach (Sylta et al., 2014), the annual training time consisted of 90–95% endurance training distributed into 85–89% LIT, 1–3% MIT, and 8–11% HIT, with the remaining being 5–10% strength and 1–3% speed training. The annual periodization of training volume with the placement of HIT blocks is presented in Figure 1 and described in more detail for one of these athletes in Solli et al. (2019). The head coach (author STS) and a physiologist (author TK) supervised most of the HIT blocks. A HIT block was typically around 8–13 days, with 5–7 of the days performed during training camps, in which a total of 7–10 HIT sessions were performed. The remainder of the training performed during a HIT block consisted of a few LIT sessions from 90 to 150 min and heavy strength sessions. During blocks, all interval sessions were effort-matched, with skiers aiming for steady-state speed during both individual intervals and throughout every 5 × 4 min session. The target intensity was 90–95% of HR_max_ during working periods, and interval speed was adjusted individually to match the effort-matched goal of the sessions. Most HIT sessions were performed as 5 × 4 min intervals with 2 to 3 minutes of active recovery (target intensity 65–75% of HR_max_) in between. Six different exercise modes were utilized during the HIT blocks. In the general preparation phase (May–October), HIT sessions were performed as flat/uphill running, uphill running with poles, and roller skiing in classic and skating techniques, while during the specific preparation phase (November–December) and competition period (January–March), HIT was performed as classic and skating XC skiing.

### Monitoring of HIT Sessions

Athletes' maximal HR (HR_max_) was tested annually by highly trained test personnel during uphill treadmill running until voluntary exhaustion. The target HIT HR was calculated as 90–95% of HR_max_. During interval training, the athletes were instructed to reach 90% of HR_max_ during the first 2 min of the first interval. In addition, each following interval should be achieved with a slightly higher HR than the previous one, ending the last interval with 2 min at 95% of HR_max_. The athletes were instructed to avoid starting at too high of an intensity and to stay disciplined within their individual target HR zone. The goal was to perform all five intervals at an equal or slightly increasing speed with proper technique. Athletes wore a polar HR monitor (Polar Electro, Finland) during interval training sessions and reported the mean HR during the last minute of each interval to the exercise physiologist. Bla^−^ concentration of 5-μL samples were taken from the fingertip and analyzed by first-generation portable Lactate Pro analyzer kits (Arkray Inc., Kyoto, Japan) directly after each 4 min interval using a capillary blood sample collected from one fingertip following standard procedures. The blood sample for measuring Bla^−^ was collected within 1 min after completing an interval, and athletes were measured one to three times during a 5 × 4 min interval session.

### Interviews

To gather information about the experiences of the performance using the BP regimen, semistructured interviews with some of the athletes, the coach, and the physiologist were conducted.

### Statistics

All data are presented as mean (SD). Shapiro-Wilk tests were used to confirm normal distribution and homogeneity of variance in all dependent variables. Thereafter, either one-way analyses of variance with Bonferroni *post hoc* test or *t*-testing was employed. A two-sided *P* < 0.05 was considered significant. For the interview data, a content analysis was conducted independently by two researchers (GSS and ØS) to categorize responses. Direct, verbatim quotes were used to exemplify the different categories.

## Results

**Figure 2:**
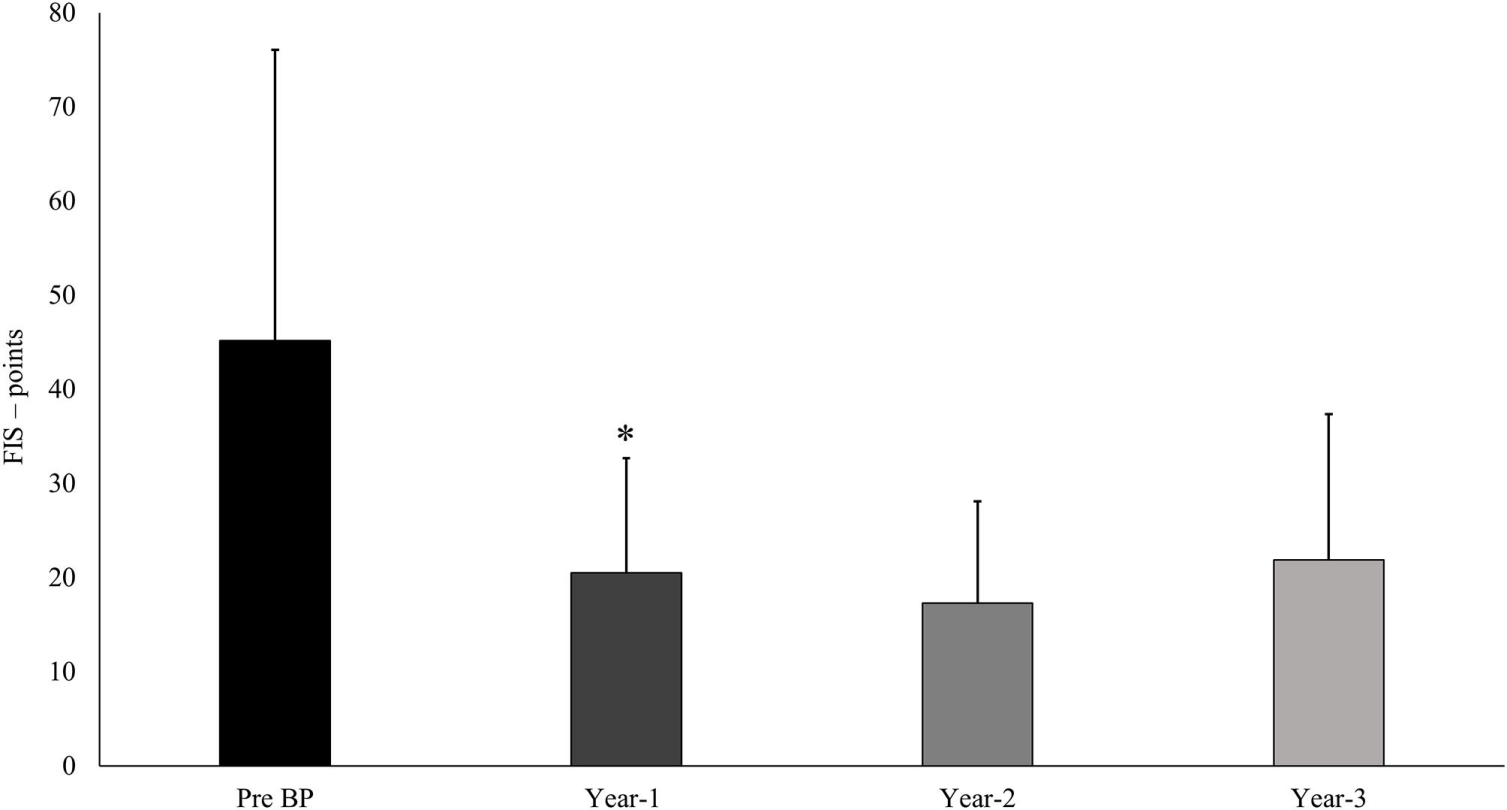
The development of performance measured in International Ski Federation's (FIS's) ranking points for the eight world-class female cross-country skiers who were included in the team and used the block periodization model (BP) in all of the three investigated seasons (year 1–3). *Significantly different from the previous season (*P* < 0.05). Pre-BP, performance during the last season before introduction of BP.

Performance analysis using the International Ski Federation's (FIS's) ranking points showed that the eight athletes using BP during all the three reported seasons significantly improved their performance during the first year (2003/2004) and maintained this performance level during the subsequent seasons (Figure 2). Similar patterns were observed in the remaining six athletes who were only part of the team for one or two seasons.

The 572 Bla^−^ and HR samples included 208 from the 2003–2004 season, 141 from the 2004–2005 season, and 223 from the 2005–2006 season in the exercise modes described in Table 1. The mean overall Bla^−^ value was 7.3 ± 2.1 mmol · L^−1^. The mean HR_max_ was 194 ± 8 beats · min^−1^. The time spent within each athlete's target HR zone (90–95% of HR_max_) ranged from 3.8 to 13.4 min (mean, 9.0 ± 4.3) out of the total HIT interval working time (~20 min). Description of HR and Bla^−^ values, including a comparison between exercise modes, is shown in Table 1. HR (both absolute and relative to maximal HR) during roller ski classic and ski classic (with large portions of double poling) was lower than the other exercise modes (*P* < 0.05). The Bla^−^ values during classic skiing were significantly lower for all exercise modes other than ski skating (*P* < 0.05).

**Table 1:** Heart rate and blood lactate concentration from 63 high-intensity interval sessions among 14 world-class female XC skiers.

	**Heart rate (beats** **·** **min**^****−1****^**)**				**Heart rate (% of max)**				**Blood lactate (mmol** **·** **L**^****−1****^**)**			
**Season**	**03/04**	**04/05**	**05/06**	**Total**	**03/04**	**04/05**	**05/06**	**Total**	**03/04**	**04/05**	**05/06**	**Total**
Number of tests	208	141	223	572	208	141	223	572	208	141	223	572
Total	177 ± 9	176 (8)	175 (9)	176 (9)	91 (3)	91 (3)	91 (3)	91 (3)	7.6 (2.2)	7.2 (2.0)	7.2 (2.0)	7.3 (2.1)
**Exercise modes**												
Running	180 ± 8	177 ± 8	175 ± 8	178 ± 8	93 ± 2	92 ± 3	91 ± 3	92 ± 3^*^^#^	7.3 ± 1.6	6.6 ± 1.8	7.5 ± 1.4	7.3 ± 1.6^#^
Running with poles	180 ± 8	177 ± 9	177 ± 6	178 ± 8^*^	92 ± 3	91 ± 3	92 ± 2	92 ± 3^#^	7.6 ± 2.0	7.8 ± 2.0	8.4 ± 1.5	7.8 ± 1.9^*^^#^
Rollerski skating	175 ± 9	175 ± 9	176 ± 8	176 ± 9	90 ± 3	91 ± 2	91 ± 2	91 ± 3^#^	7.4 ± 2.4	7.8 ± 2.2	7.8 ± 2.5	7.7 ± 2.4^#^
Rollerski classic	176 ± 7	175 ± 8	173 ± 8	175 ± 8	91 ± 2	90 ± 4	90 ± 3	90 ± 3	9.1 ± 2.5	6.2 ± 1.4	6.6 ± 1.7	7.1 ± 2.1^#^
Ski skating	164 ± 7	179 ± 7	176 ± 11	176 ± 10	90 ± 4	92 ± 3	93 ± 2	92 ± 3^*^^#^	7.8 ± 2.7	8.0 ± 2.2	6.0 ± 1.4	7.0 ± 2.1
Ski classic	173 ± 7	175 ± 7	176 ± 17	175 ± 11	89 ± 3	89 ± 3	89 ± 5	89 ± 4	6.1 ± 2.0	5.9 ± 1.5	5.8 ± 1.6	5.9 ± 1.7

*Data are reported as mean and standard deviation (SD) and include the mean of each season between 2003 and 2006 and values for each season (03/04, 04/05, and 05/06). Statistical testing was performed between seasonal means*.

* *P < 0.05 vs. rollerski classic*,

# *P < 0.05 vs. ski classic*.

A total of 297 interval working periods had no record of an interval number (1–5) when the physiological measurements were done (HR, 175 ± 9 beats · min^−1^; %HR_max_, 91 ± 3%, and Bla^−^ 7.1 ± 2.0 mmol · L^−1^). For the measurements including recordings of interval working period numbers during physiological monitoring, HR and Bla^−^ increased from interval number 1–3 and further from 3 to 5 (*P* < 0.05).

Comparisons of the first two (150 measurements) and two final (102 measurements) HIT sessions during each HIT block revealed no difference in absolute HR (176 ± 9 and 175 ± 8 beats · min^−1^), but significantly lower values were noted relative to HR_max_ (91 ± 3% and 90 ± 2% of HR_max_) and significantly higher Bla^−^ (7.6 ± 2.2 and 8.2 ± 2.0 mmol · L^−1^) (*P* < 0.05).

**Figure 3:**
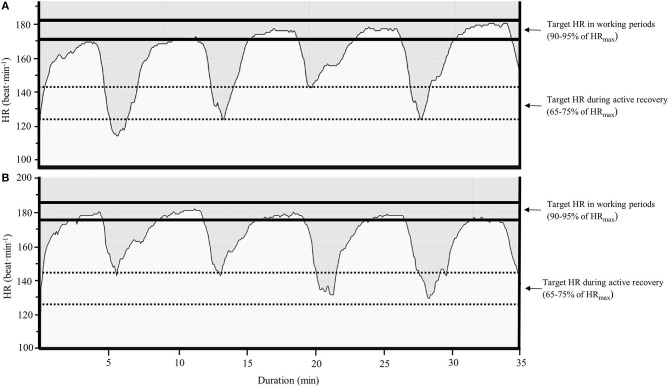
The development of heart rate during a successfully **(A)** and unsuccessfully **(B)** 5 × 4-min high-intensity interval (HIT) training session, performed by two different athletes during a HIT block.

Interviews with the athletes, the coach, and the physiologist highlighted the importance of being physically and mentally prepared before entering the HIT block, intensity control, recovery routines, and individualization of interval training (**Table 3**). Figure 3A illustrates a successfully performed 5 × 4 min interval training with correctly targeted HR development, with Figure 3B illustrating a less optimal session. The negative HR development illustrated in Figure 3B was a typical pattern in poorly recovered athletes or athletes starting the sessions at too high of an intensity/speed.

## Discussion

This article describes the physiological responses of HIT sessions during a long-term BP of HIT regimen by comparing the HR and Bla^−^ response utilizing different exercise modes, the development of HR and Bla^−^ within interval sessions, and the physiological response during the first and final sessions within the HIT blocks. The main findings were as follows: (1) the average intensity of interval sessions was 91% of HR_max_ with corresponding Bla^−^ values of 7.3 m mmol · L^−1^; (2) similar HR and Bla^−^ values were observed in the different exercise modes, except for a lower HR being observed in classic (roller skiing and on snow), including large portions of double poling, and lower Bla^−^ values being observed in on-snow classical skiing compared to the majority of the other modes; and (3) there was a trend for a progressive increase in HR and Bla^−^ values from the first to the fifth interval, with a similar pattern in Bla^−^ values when comparing the two first and the two final interval sessions in the HIT blocks, indicating that the athletes managed to perform the HIT sessions at target intensity throughout the HIT blocks and as planned with a slight increase in workload from interval 1 to 5.

The high density of HIT sessions, with short recovery time between sessions and subsequent risk of accumulation of fatigue from sessions targeting the same systems, is the main challenge in BP regimens. In this case, maintenance of a high training quality throughout the HIT blocks was regarded as crucial, and data indicate that physiological responses maintained the same throughout each block. The positive performance development (measured in FIS points), which was significant in the first year of using BP, indicates that also the chronic adaptations were positive. This coincided a positive VO_2max_ response among most of the athletes, and although these values were not accessible for analysis, the coach and the physiologist reported an increase in the range of 5–15% in VO_2max_.

To achieve high-quality HIT sessions during the HIT blocks, all sessions were closely supervised with a focus on an even or slightly increased speed from the start to the end of the sessions, allowing HR to gradually approach the target intensity zone of 90–95% of HR_max_ (Karlsen et al., 2017). Our data (Table 2) indicate that the athletes successfully achieved the aim of progressively increasing intensity within their target HR zone. Active athlete control of HR during sessions was supported with systematic Bla^−^ measurements. The average intensity of HIT sessions was 91%, with the intensity increasing from 90 to 93% from the first to the final interval, indicating high training discipline adhering to the training system and targets. As previously suggested by Solli et al. (2019), this precise steering of intensity during HIT sessions likely contributed to reduced recovery time, thereby increasing the athlete's ability to perform the high frequency of HIT sessions at a high quality and tolerate the overall training load during blocks. In fact, many of the athletes experienced being in better physical shape during the second HIT session of the day (first sessions started between 8 and 9 a.m., and the second session between 4 and 5 p.m.). It is uncertain if this was due to a physiological response of session 1, sleep patterns, or circadian rhythms. However, this was only the case if the first session was not performed at too high of an intensity.

**Table 2:** Heart rate and blood lactate concentration at intervals 1–5 among 14 world-class female XC skiers (mean data from 2003 to 2006).

**Interval working period (1–5)**	**No. samples**	**Heart rate (beats · min^**−1**^)**	**Heart rate (% of max)**	**Blood lactate (mmol · L^**−1**^)**
1	64	174 ± 9^*^^#^	90 ± 3^*^^#^	6.5 ± 1.8^*^^#^
2	64	177 ± 8	91 ± 3^#^	7.3 ± 2.0^#^
3	54	178 ± 9	92 ± 2	7.7 ± 1.9^#^
4	49	180 ± 9	92 ± 2	7.9 ± 1.9
5	44	180 ± 8	93 ± 3	9.0 ± 2.2

*Data are reported as mean ± standard deviation (SD) of data collected between 2003 to 2006*.

* *P < 0.05 vs. interval number 3 and 4*,

# *P < 0.05 vs. interval number 5*.

Most studies showing beneficial adaptation using BP of HIT are performed on cycle ergometers or treadmills, making the intensity control easier. During “real-life” training camps, as done here, with highly competitive athletes using different exercise modes and challenging hilly terrains, the intensity control was more challenging. The two different HR curves illustrated in Figure 3 clearly show the difference between a high-quality HIT session with a progressive increase in HR (3A) and a session showing a downward curve (3B) with too early of an accumulation of fatigue. The latter curve was typically seen in fatigued athletes or in athletes starting at too high of a pace for the first interval. The team physiologist continuously supported the athletes with information about their intensity control to educate them to perform high-quality sessions and reach their individual HR and Bla^−^ targets. Furthermore, the team had a high focus on recovery routines and illness prevention (nutrition, change of clothes after sessions, hygiene, etc.) during the HIT blocks and ensured that sufficient recovery was included after each block. Altogether, these aspects might have helped the team to tolerate the training and to stay healthy, as the number of injury and illness incidents did not increase during or after these camps, compared to other training periods.

The athletes highlighted the importance of reducing the training load before HIT blocks in order to be both physically and mentally prepared to perform. Another important point that likely allowed the skiers to tolerate and respond positively to such a high number of HIT sessions is the alternation between several exercise modes, in which these well-trained XC skiers attained relatively similar HR and Bla^−^ values. This is likely possible because they were highly trained and used to performing HIT sessions in all these modes. During the HIT blocks in the current study, participants utilized six different exercise modes, including specific (classic and skating techniques on skis or roller skis), semispecific (running with poles), and non-specific (running) exercise modes. The different muscle recruitment patterns in the various exercise modes could have theoretically affected the HR and Bla^−^ responses of each exercise mode, as they activated the upper and lower body to a different extent. However, only classical on-snow and roller skiing with large amounts of double poling induced slightly lower HR values during intervals. Lower HR values during upper-body dominant exercises compared to lower-body exercises have been previously reported (Borg et al., 1987; Koppo et al., 2002; Holmberg et al., 2006, 2007), and a previous study reported a 13% lower HR_peak_ during upper-body poling with fixed legs compared to running (Undebakke et al., 2019). However, the HR relative to peak HR in the given mode did not differ between exercise modes (Undebakke et al., 2019), and it is therefore likely that the observed differences in %HR_max_ in classical (roller) skiing would diminish if the HR values were normalized for peak HR in double poling. Furthermore, lower Bla^−^ values were observed in on-snow classical skiing compared to several other exercise modes. Most on-snow classical HIT sessions were performed at the start of the competition season, and thus, more competition-like terrain was used. Therefore, these sessions included steeper uphill terrain with subsequently increased use of the diagonal stride technique (Solli et al., 2018), where lower Bla^−^ values for a given %HR_max_ are reported compared to upper-body poling, although similar Bla^−^ values were observed in running (Undebakke et al., 2019).

This microperiodization of different exercise modes, with differential loading of the upper and lower body, is likely essential in maintaining the quality of sessions, as well as avoiding muscular fatigue throughout the HIT block (Solli et al., 2019). In this publication, we provide novel data on HR and Bla^−^ values during effort-matched 5 × 4 min intervals in different exercise modes utilized by XC skiers. The effort-matched goal of the session was adjusted individually to match the athletes target intensity of 90–95% of HR_max_ during working periods. Although focus on target intensity might have influenced the comparison of HR and Bla^−^ between exercise modes, we found HR and Bla^−^ to correspond well across exercise modes in these elite endurance athletes. Therefore, the reference data of HR and Bla^−^ across intervals and between exercise modes, together with the practical experiences for achieving successful interval sessions (Table 3), are relevant information for coaches and athletes in the planning and execution of BP training regimens and for further scientific studies of HIT. Future studies should examine the peak and threshold values for HR and Bla^−^ for each exercise mode, and microperiodization of HIT blocks, with utilization of various exercise modes, also requires further attention.

**Table 3:** Overview of the focus areas and experiences of the athletes, the coach, and the physiologist during three annual seasons using block periodization of high intensity training.

**Categorization**	**Athletes**	**Coach**	**Physiologist**
**Focus areas before under and after HIT blocks**			
Be mentally and physically prepared	Reducing the training load to be physically and mentally prepared for the HIT block. Mentally demanding to perform the HIT−blocks, important to be in a rested state before and provide enough rest after each block.	Ensuring that athletes are healthy and have reduced their training load before starting the HIT block.	Ensuring that each athlete had tested their HR_max_ and emphasize the knowledge of target HR zone, HR development during sessions, and knowledge of expected Bla^−^ response to exercise mode and HR performance.
Intensity steering	Follow my own pace, better to start the session at too low than too high speed. I often felt in better shape at the second HIT session of the day. However, if the intensity was too high on the first session, this was not the case.	Close supervision that each athlete follows his own target HR zone. Highlighting technical tasks during the specific sessions and dampening the competitive aspect of the sessions. Choose the right terrain for the specific session, making it easier for the athletes to perform the session at the target intensity.	HR and Bla^−^ were recorded during HIT sessions to increase exercise intensity control, most often reducing and controlling exercise speed, rarely with the need for increasing it in these highly competitive athletes. HR curves from successfully performed sessions used as illustrations, both in new athletes as well as repetition throughout the seasons.
Recovery routines	Proper rest between sessions, often including 1–2 h sleep.	Reducing the length of all additional training (LIT and strength sessions) during the HIT blocks.	Following up recovery routines (nutrition, hydration, sleep, change of clothes after session, etc.) to avoid illness in athletes, coaches, and the support team. Specific routines in connection with altitude camps.
Individualization	Sometimes difficult to follow your own pace, being at a training camp with competing athletes.	Making individual plans for the HIT blocks adjusting the duration and number of HIT sessions. The most experienced athletes performed a higher number of HIT session during blocks.	Follow up the individual intensity focus in each athlete, with measurements of Bla^−^ and HR. With an evaluation after each session, focus on educated guessing of HR and Bla^−^.

A recent meta-analysis (Mølmen et al., 2019) reported evidence for beneficial short-term effects of BP compared to TRAD regarding VO_2max_ and maximal power output development in trained athletes, highlighting that BP of HIT could serve as an adequate, alternative strategy with potentially higher training effects than TRAD. However, the long-term adaptations following BP of HIT is currently not well-understood and requires further examination. In a similar pattern as indicated here, the case study of the world's best XC skier (Solli et al., 2019) indicated that BP of HIT had the greatest benefits in the first 2 years, whereas a stagnation was observed in the following seasons. In addition, she had a similar level of success with TRAD (Solli et al., 2017, 2019). Thus, the performance improvement during the initial years indicates that BP of HIT with careful control of intensity and progress could be especially beneficial in some periods of endurance athletes' careers, whereas other models could be more beneficial when the potential of such a method has been reached.

### Methodological Considerations

The main strength of this publication is the high external validity, allowing us to provide unique data from systematic field testing of elite female endurance athletes during several seasons of extensive BP of HIT. Although we could track the performance progress during these three seasons, a main limitation to the publication is the lack of physiological test data (e.g., VO_2max_, anaerobic threshold and work economy) and external load monitoring during the training sessions. However, the publication does not report on a designed exercise intervention, but routine field testing, and because VO_2max_ testing was done more than 10 years ago by external personnel, we did not have access to these data at the time of publishing. In addition, external load measurements are rarely used in XC skiing because most of the sessions are performed across undulating terrain at varying external conditions. However, global navigation satellite system tracking will soon allow for studies to provide valid information about external load also in XC skiing.

### Practical Implications

Based on the experiences from the current long-term BP training regimen, we suggest the following principles as key to success:

1. Individual intensity control during HIT sessions, where all athletes are made familiar with their target intensity HR zone and ways to progressively reach this. Support HR control with systematic Bla^−^ measurement if feasible.
2. Alternate between different exercise modes and terrains to allow for variability in the muscular load during HIT sessions.
3. Make individual adjustments of HIT loads during blocks, and reduce the volume of LIT, MIT, and strength training.
4. Reduce the training load before HIT blocks to be mentally and physically prepared.
5. Maintain a high focus on recovery routines during HIT blocks.
6. Reduce the training load after HIT blocks to enhance recovery and induce adaptation.

## Conclusion

This study illustrates how long-term, HIT-block periodization was used among world-class XC skiers, in which target HR and Bla^−^ levels were achieved in all the six exercise modes employed. As key to successful blocks, both athletes and coaches highlighted the importance of controlled intensity steering to allow high-quality HIT sessions throughout the entire HIT block.

## Data Availability Statement

The data analyzed in this study is subject to the following licenses/restrictions. The dataset presented in this article may be obtained by contacting the corresponding author. Requests to access these datasets should be directed to Trine Karlsen, trine.karlsen@nord.no.

## Ethics Statement

Ethical review and approval was not required for the study on human participants in accordance with the local legislation and institutional requirements. The patients/participants provided their written informed consent to participate in this study.

## Author Contributions

SS and TK planed, performed, and recorded the testing and training reported in the study. GS contributed data to the study as an athlete. TK, GS, SS, and ØS analyzed and presented data, authored, finalized the manuscript for publication, and approved the final manuscript. All authors contributed to the article and approved the submitted version.

## Conflict of Interest

The authors declare that the research was conducted in the absence of any commercial or financial relationships that could be construed as a potential conflict of interest.

## Abbreviations

BP: block periodization
HIT: high-intensity training
MIT: moderate-intensity training
LIT: low-intensity training
TRAD: traditional periodization model
XC: cross country.
